# Fast Measurement of Magnetostriction Coefficients for Silicon Steel Strips Using Magnetostriction-Based EMAT

**DOI:** 10.3390/s18124495

**Published:** 2018-12-19

**Authors:** Weiping Ren, Ke Xu, Peng Zhou

**Affiliations:** 1 Collaborative Innovation Center of Steel Technology, University of Science and Technology Beijing, Beijing 100083, China; ping28198@126.com; 2 National Engineering Research Center of Advanced Rolling Technology, University of Science and Technology Beijing, Beijing 100083, China; zhoupeng@nercar.ustb.edu.cn

**Keywords:** magnetostriction coefficient measurement, electromagnetic acoustic transducer (EMAT), silicon steel, Lamb wave

## Abstract

Strain gauges and optical methods are commonly used to measure the magnetostriction coefficient of a sample. All these methods require a specific size sample and can only realize offline measurement, which is time-consuming. Therefore, we propose a new method using a magnetostriction-based electromagnetic acoustic transducer (EMAT) to measure the magnetostriction coefficient. The amplitude of the ultrasonic waves generated by the EMAT is applied to characterize the magnetostriction coefficient of a sample. A nonlinear magnetostriction finite element model is established, and the simulation results show that the amplitude of the ultrasonic wave generated by the magnetostriction-based EMAT is proportional to the magnetostriction coefficient of the material. Experiments are carried out on silicon steel strips with different silicon contents. The results show that the method can effectively measure their relative magnetostriction coefficients. Furthermore, the structure of the magnetostriction-based EMAT is optimized to maximize efficiency. The excitation and receiving transducers reach their maximum efficiency when the static magnetic flux densities are 3.5 mT and 6.8 mT, respectively. Moreover, the relative error caused by the vibration reaches the minimal size when the lift-off of the receiving coil is set to 3 mm around. This method is fast and can be applied to online measurement.

## 1. Introduction

It is always desirable to keep the magnetostriction coefficient of electrical steel as low as possible to reduce the operating noise of the transformer or motor [1]. Generally, the magnetostriction coefficient of electrical steel can be reduced by increasing the silicon content [2]. For electrical steel manufacturers, it is usually necessary to sample and measure their products to ensure that their magnetostriction coefficients meet the requirements.

A conventional method for measuring the magnetostriction coefficient is to place the sample in a dynamical uniform magnetic field and measure the strain using various equipment, such as a strain gauge [3], laser Doppler vibrometer [4], laser displacement meter [5], or digital holographic microscope [6]. A two-dimensional (2-D) model based on a mechanical elasticity analogy is also proposed to calculate the magnetostriction of electrical steel [7]. However, these methods are generally inefficient and require destructive sampling for the larger sized specimens. For common electrical steels, it is difficult to achieve an accurate measurement through the strain gauge method due to their low magnetostriction coefficients, while the optical methods are complicated and their repeatability is poor.

**Figure 1:**
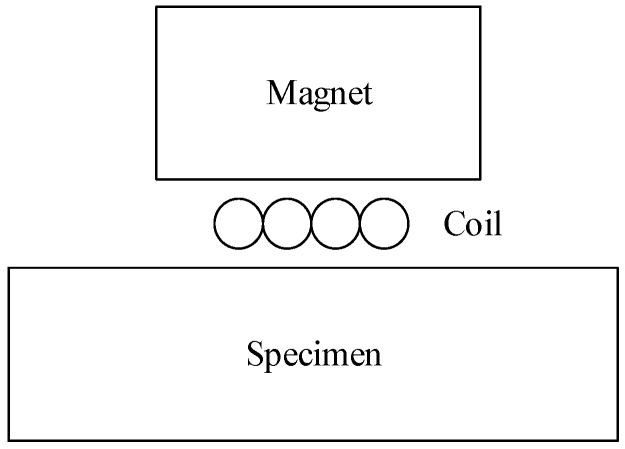
Typical configuration diagram of an electromagnetic acoustic transducers (EMAT).

A new measurement method is thus proposed, which uses magnetostriction-based electromagnetic acoustic transducers (EMATs) to measure the magnetostriction coefficients of silicon steel strips and can realize fast online measurement. EMATs are electromagnetically coupled ultrasonic transducers which can generate and detect ultrasonic waves on electrically conducting media [8,9,10]. EMATs are widely used because of their fast detection speed and non-contact characteristics, for example, in the defect detection or thickness measurement in metal pipes, plates, bars, rails, and so on [11,12,13,14]. A typical EMAT generally includes a sample, a bias magnetic field, and an excitation coil, as shown in Figure 1. When a ferromagnetic material such as steel acts as the sample, the conversion mechanism of the EMAT is contributed by various mechanisms, including the Lorentz force mechanism, magnetostriction mechanism, and magnetization force mechanism [8,9,15]. Among them, the Lorentz force mechanism is considered to be the main mechanism [16,17]. Lorentz forces are not very sensitive to the magnetostriction coefficient of materials, although they are sensitive to conductivity [18]. Only the magnetostriction mechanism is sensitive to changes in the magnetostriction coefficient of the material. 

**Figure 2:**
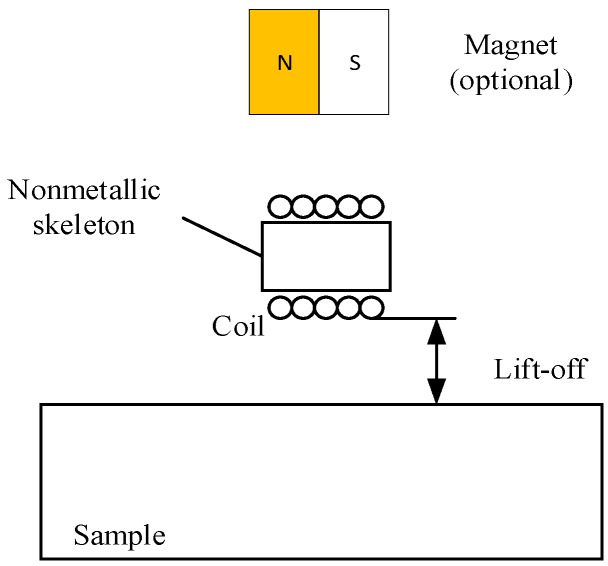
Configuration diagram of a magnetostriction-based EMAT.

A specific EMAT based on the magnetostriction mechanism was proposed in a previous study [19], and its configuration is shown in Figure 2. Compared with traditional transducers, the magnetization-based EMAT’s excitation coil is placed at a considerable distance from the sample. Moreover, the horizontal bias magnetic field is much smaller than that in the traditional transducer. Once the lift-off of the excitation coil is greater than 7 mm, the magnetostriction mechanism becomes the main contributor, and the other two mechanisms can be ignored [19]. Therefore, at a fixed lift distance greater than 7 mm, the transducer’s conversion efficiency will be primarily dependent on the magnetostriction coefficient of the sample. Individually, the sample’s larger magnetostriction coefficient leads to a higher conversion efficiency of the transducer, that is, to a higher amplitude of the ultrasonic wave, and vice versa. Therefore, it is conceivable to evaluate the magnetostriction coefficient of the material by the magnitude of the ultrasonic wave.

The feasibility of the magnetostriction-based EMAT for measuring the magnetostriction coefficient is studied. The optimal configuration of the transducer for measuring 0.3-mm thick silicon steel strips is determined, including the permanent magnet lift distance and the receiving coil lift distance.

## 2. Simulation

**Figure 3:**
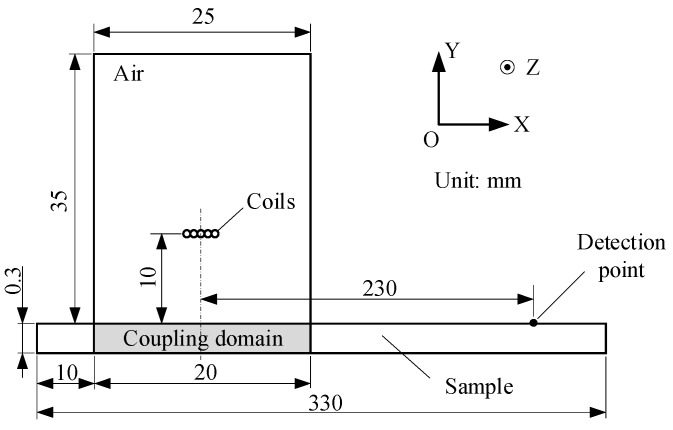
The geometry of the two-dimensional (2-D) finite element (FE) model used to simulate the ultrasonic generation by the magnetostriction-based EMAT.

Whether or not the amplitude of the ultrasonic wave can characterize a material’s magnetostriction coefficient is contingent on whether there is a one-to-one correspondence between them. In this section, finite element simulation is employed to reveal their relationship. Concretely, a nonlinear 2-D finite element (FE) model is built using the multi-physics simulation software COMSOL Multiphysics 5.3^®^ to simulate the excitation process of the magnetostriction-based EMAT. The geometry of the model is shown in Figure 3, which includes air, coil, and sample domains. Because the static bias field is optional and only applied to enhance the conversion efficiency, no bias magnetic field is added to the model. Both the left and right ends of the sample are set to a low reflection boundary to reduce the influence of the reflected wave on the result.

Silicon steels usually include oriented silicon steel and non-oriented silicon steel. The magnetostriction of the oriented silicon steel is anisotropic. The non-oriented silicon steel’s magnetostriction is generally considered to be isotropic, although slight directionality may be introduced by rolling or other processes [20]. The silicon steel’s magnetostriction coefficient rises with the increment of the magnetic flux density until it reaches saturation, called saturated magnetostriction $\lambda_{s}$. Moreover, the magnetostriction coefficient of silicon steel has an approximately linear relationship with the magnetic flux density [5]. Magnetic flux density is contributed by magnetization and magnetic field. Generally, the magnetization is the main contributor to the magnetic flux density for the silicon steel. So, it is assumed that the magnitude of magnetization has a linear relationship with the magnetostriction coefficient. A magnetostriction model coupling magnetization to strain is established. The constitutive equation is:

(1) $$\varepsilon_{me}=\frac{3}{2}\frac{\lambda_{s}}{M_{s}^{2}}dev\left(T\right)$$

where T is a tensor composed by the magnetization vector M, and dev(T) is a deviatoric tensor of T, used to ensure volume invariance. $M_{s}$ is the saturated magnetization, and $\lambda_{s}$ is the saturated magnetostriction coefficient. The current through the coil creates dynamic magnetic fields and eddy currents in the sample, which in turn produce a dynamic Lorentz force. The previous study showed that the Lorentz force can be neglected at the current configuration, and the magnetostriction dominates the conversion mechanism [19]. Therefore, the model does not consider the contribution of the Lorentz force mechanism.

**Figure 4:**
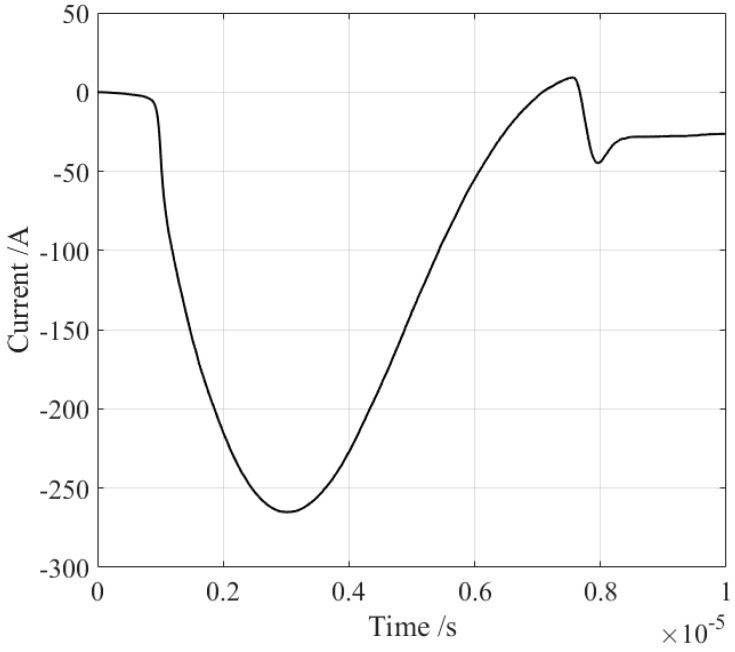
The current pulse flowing through the excitation coil.

Magnetostriction coupling is applied only to a local region directly under the coil to speed up the calculation. The material of the sample is set to 35PN300 silicon steel. The magnetization curve of this material is from COMSOL’s built-in library, its conductivity is 1.96 MS/m, its Young’s modulus is $2\times 10^{11}\;\mathrm{Pa}$, its Poisson’s ratio is 0.26, and its density is 7.65 $\mathrm{kg}/m^{3}$. The copper coil has five turns, and the diameter of the wire is 0.68 mm. The current flowing into the coil is derived from the measurement results of the actual EMATs, as shown in Figure 4, which is a broadband pulse current with a maximum current of approximately 270 A and a duration of several microseconds. The sample’s saturated magnetostriction coefficient $\lambda_{s}$ is attenuated from $18\times 10^{-6}$, and the attenuation coefficients are 1.0, 0.8, 0.6, 0.4, and 0.2. In general, materials with different magnetostriction coefficients are also accompanied by differences in other electromagnetic parameters, such as the electrical conductivity and Young’s modulus, which is addressed further in the Discussion section.

Lamb waves and shear horizontal (SH) waves are two types of ultrasonic wave modes excited by the EMAT in the plate [21]. The EMAT configuration in our experiments can generate multimode Lamb waves, which are separated into two groups of modes—symmetric (S) and anti-symmetric (A)—that have complex but well-understood dispersion curves [22]. The S0 mode and A0 mode Lamb waves are generated in the experiments as we are operating at a relatively low frequency thickness product, below the cut-off of higher order modes. 

**Figure 5:**
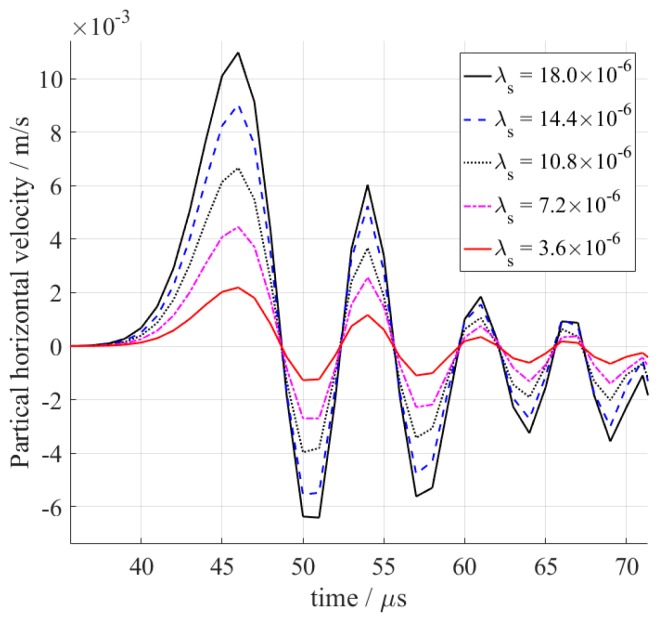
Received S0 Lamb waves with different magnetostriction coefficients.

**Figure 6:**
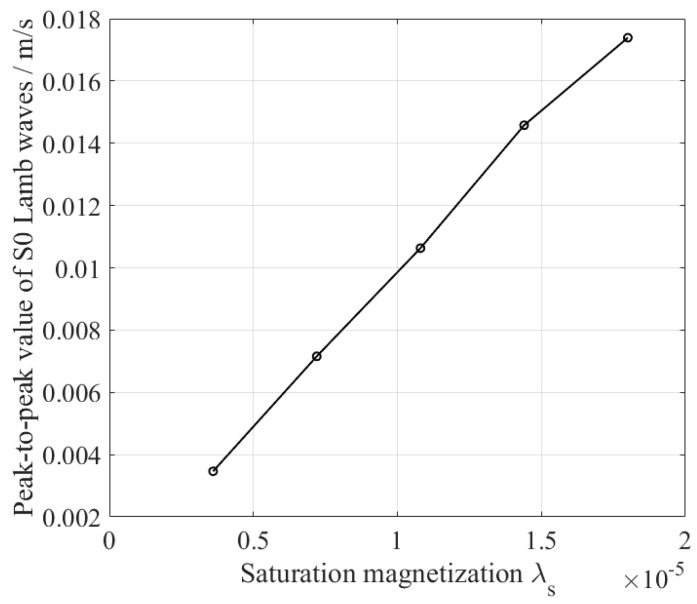
The relationship between the amplitude of S0 Lamb wave and the magnetostriction coefficient of the sample.

The velocity of the S0 wave is always higher than the A0 wave; that is, the S0 wave arrives ahead of the A0 wave. Therefore, the S0 wave is treated as a reference to minimize the superposition of reflected waves. A checkpoint is set up on the surface of the specimen 230 mm away from the excitation coil to represent the receiving EMAT. A receiving EMAT detects the vibration velocity of the particle [18]. So, the horizontal velocities of the checkpoint are recorded as a function of time. The S0 Lamb waves under different magnetostriction coefficients are shown in Figure 5. The amplitude of the S0 Lamb wave decreases as the magnetostriction decreases. For comparison, peak-to-peak values of these S0 Lamb waves are calculated and drawn in Figure 6. The results show that the conversion efficiency of the magnetostriction-based EMAT is indeed proportional to the magnetostriction coefficient of the sample. It is feasible to characterize the magnetostriction coefficient of the material by the amplitude of the guided waves.

## 3. Experiments

Three non-oriented silicon steel strip samples with different magnetostriction coefficients were prepared. They had the same elemental composition except silicon, and their silicon contents are 3.0%, 4.5%, and 6.0%, respectively. All the samples have the same size—a width of 38 mm, a length of 500 mm, and a thickness of 0.3 mm, with the rolling direction along the length. In the experiments, the magnetostriction-based EMAT was applied to measure the magnetostriction coefficients of these samples. 

**Figure 7:**
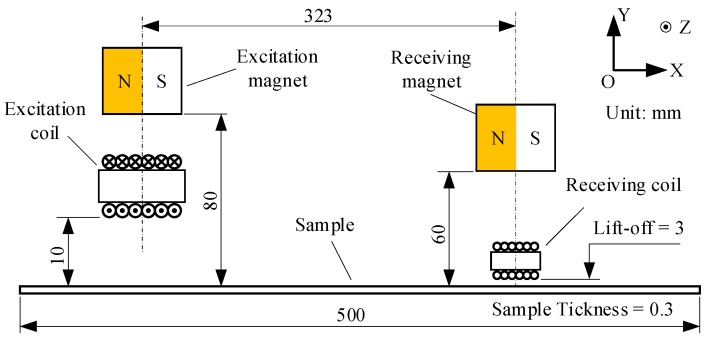
Schematic diagram of the experiment configuration.

An experiment configuration was designed as shown in Figure 7. Two separate transducers were employed as the generator and receiver. Both transducers possessed a similar configuration, but the inverse mechanism. The generator was based on the magnetostriction mechanism, while the receiver was based on piezomagnetism. NdFeB acted as the material of both the excitation and the receiving magnets, which are magnetized horizontally. Both the two magnets had the same size, which is 35, 38, and 38 mm in the X, Y, and Z directions. The excitation coil was wound from enameled copper wire with a diameter of 0.68 mm and 10 turns, and its size in the X, Y, and Z directions was 6.8, 21, and 38 mm with a lift-off of 10 mm. The wire diameter of the receiving coil was 0.1 mm with 26 turns. The size of the receiving coil was 2.6, 9, and 40 mm in the X, Y, and Z directions, and the lift-off was 2 mm. The excitation coil and the receiving coil were wound on two plastic skeletons. The pulse current flowing through the excitation coil was the same as that shown in Figure 4, and its direction was invariant. The dynamic magnetic field generated by the coil superposed the static magnetic field near the sample. These two magnetic fields need to be in the same direction. Otherwise, the opposite directions may cause the superimposed magnetic field to cross zero point, which may induce frequency distortion of the ultrasonic wave. All three samples were tested under the same configuration.

**Figure 8:**
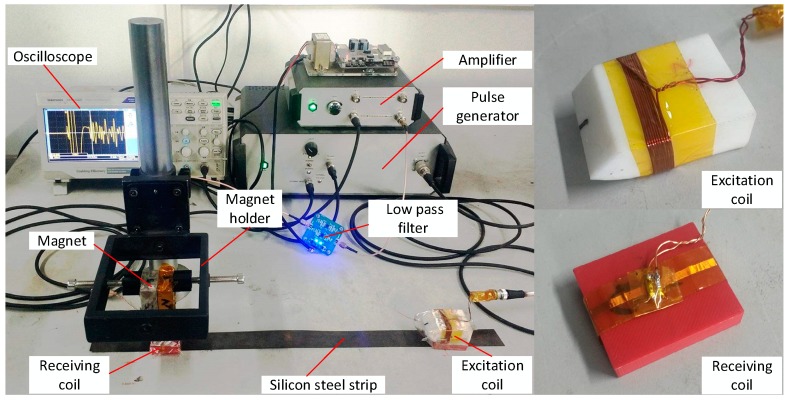
Instrumental setup.

The instrumental setup is shown in Figure 8, including a pulse generator, an EMAT generator, an EMAT receiver, a signal amplifier, a low-pass filter, and an oscilloscope. The pulse generator produced a wide-band pulse current, and its working principle involved first charging an internal high-frequency capacitor and then releasing the charge in a very short time to form a pulse current. An ultra-low noise signal amplifier was used to amplify the received signal with a gain of about 80 dB. A low pass filter was applied to filter out high-frequency noise signals (higher than 1 MHz). A holder helped to fix the magnet and adjust the distance from the magnet to the sample. An oscilloscope (Tektronix, TBS 1102B) acted as the signal collector, which could be replaced by a dedicated signal acquisition and analysis system for automatic measurement.

**Figure 9:**
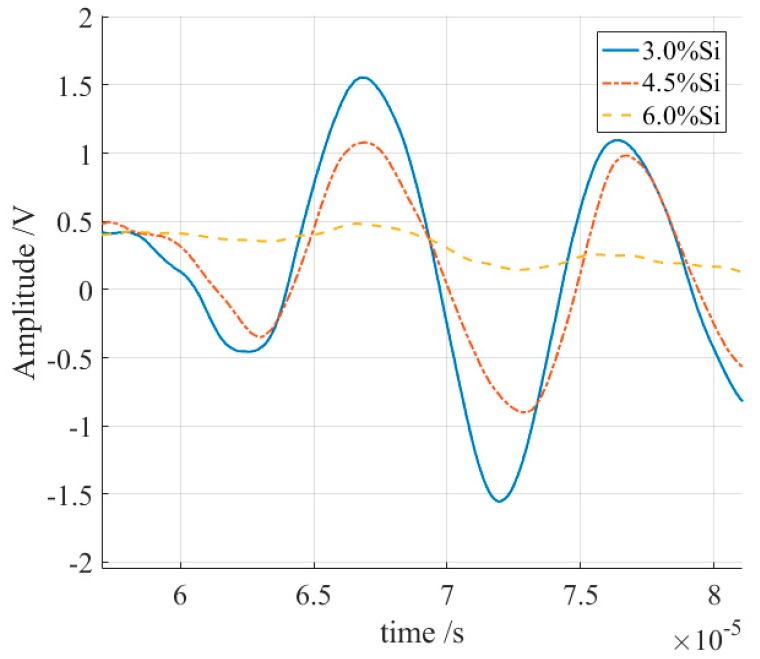
The S0 Lamb waves of the samples with different silicon contents.

**Figure 10:**
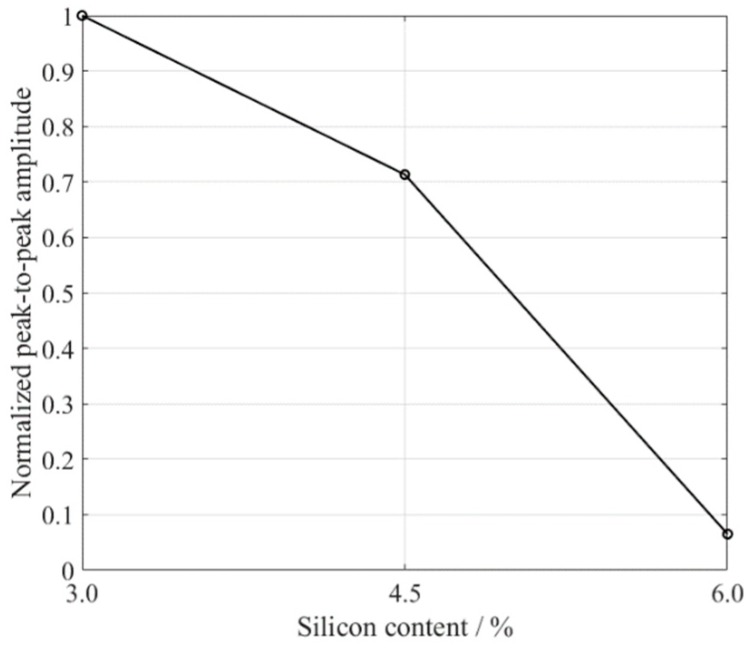
The peak-to-peak amplitude of the S0 Lamb wave versus the silicon content (normalized).

The signals from the receiving transducer were recorded and averaged over 32 times to reduce any electrical noise, and the S0 Lamb waves are shown in Figure 9. The amplitudes of these S0 Lamb waves were different. For ease of comparison, the peak-to-peak values of these S0 waves were calculated. The peak value at 66 μs was subtracted from the valley value at 72 μs as the peak-to-peak value of the S0 waves, and the normalized results are shown in Figure 10. The peak-to-peak value of S0 Lamb wave decreased with the increment of the silicon content, which indicates the decline of the magnetostriction. The magnetostriction coefficient of the sample with 6% silicon content was an order of magnitude lower than that of the sample with a silicon content of 3%, which is in accordance with the findings of others [23]. This method is able to obtain a relative measurement result. Furthermore, necessary calibration can help to determine the real magnetostriction coefficient.

## 4. Optimization

The efficiency of an EMAT is much lower than that of a piezoelectric ultrasonic transducer due to its complicated mechanism [24,25]. In order to obtain a higher signal-to-noise ratio (SNR), it is usually required to optimize the transducer [26,27,28]. The magnetostriction-based EMAT has proved to be useful in measuring the magnetostriction coefficients of silicon steel strips. The transducer’s structure needs to be optimized to improve efficiency because a higher efficiency could help to reduce the impact of electrical noise on the results. For the traditional EMAT, a higher efficiency could be achieved by enhancing the static magnetic field strength [29]. Moreover, the lift-off of the coil also has a significant influence on the conversion efficiency [30]. So, the lift-off values of both the permanent magnet and the coil were studied to elucidate their relationship with the EMAT efficiency. 

The excitation transducer and the receiving transducer each has a permanent magnet. Firstly, the relationship between the magnet’s lift-off and the transducer’s efficiency was studied by experiments. The experimental configuration diagram is shown in Figure 7. The silicon steel strip with 3.0% silicon content acted as the sample which was fully demagnetized in advance. In the experiments, the step change was only applied to the lift-off of the excitation magnet or the receiving magnet, and one of them was changed when the other was fixed. In order to reduce the effect of hysteresis, the lift-off of the magnet was gradually reduced from 220 mm so that the magnetization of the sample was gradually strengthened rather than subjected to the demagnetization process.

**Figure 11:**
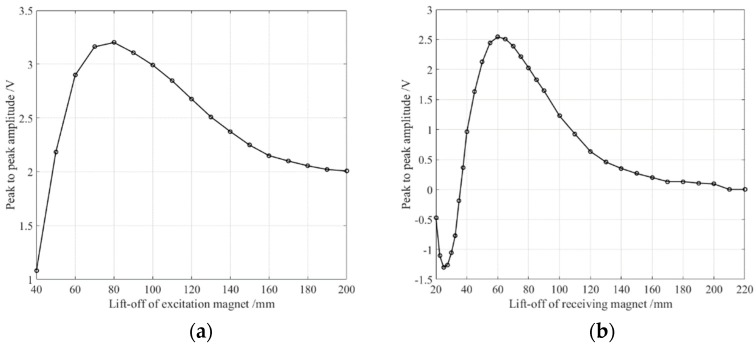
The peak to peak amplitude of the S0 Lamb wave versus the lift-off the excitation and receiving magnets. (**a**) shows the S0 wave’s peak to peak value varies with the excitation magnet’s lift-off. (**b**) shows the S0 wave’s peak to peak value varies with the lift-off of the receiving magnet.

The S0 waveforms obtained in the experiment are not shown here. Their peak-to-peak values were also treated as references. The results are shown in Figure 11, in which Figure 11a shows the relationship between the lift-offs of the excitation magnet and the S0 wave’s peak-to-peak values, and Figure 11b gives the correlation between the receiving magnet’s lift-offs and the S0 wave’s peak-to-peak values. The smaller the lift-off is, the closer the permanent magnet to the sample, that is, the higher the horizontal magnetic field strength. It should be noted that the peak-to-peak value of the S0 wave was not always observed increase as the excitation magnet’s lift-off decreases, but rose first and then fell, reaching a maximum at about 80 mm. This means that, in the silicon steel strip, the transducer obtains the highest efficiency at a mezzo horizontal bias magnetic field strength, which is quite unconventional compared to the traditional EMAT [29,31], as well as different from the response of the low-carbon steel sample [19]. The optimum lift-off for the receiving magnet was 60 mm, which is incompatible with the excitation magnet. It is interesting that the amplitude of the S0 wave’s amplitude reached zero when the lift-off of the receiving magnet was set to about 35 mm. When the lift-off was greater than 35 mm, the phase of the S0 wave was opposite that when the lift-off was less than 35 mm. One possible reason for this is that the piezomagnetism mechanism performs a major role under the low level static magnetic field, while the Faraday electromagnetic induction law accounts for the main contribution under a higher level static magnetic field. This phenomenon requires further research which is beyond the scope of this article.

**Figure 12:**
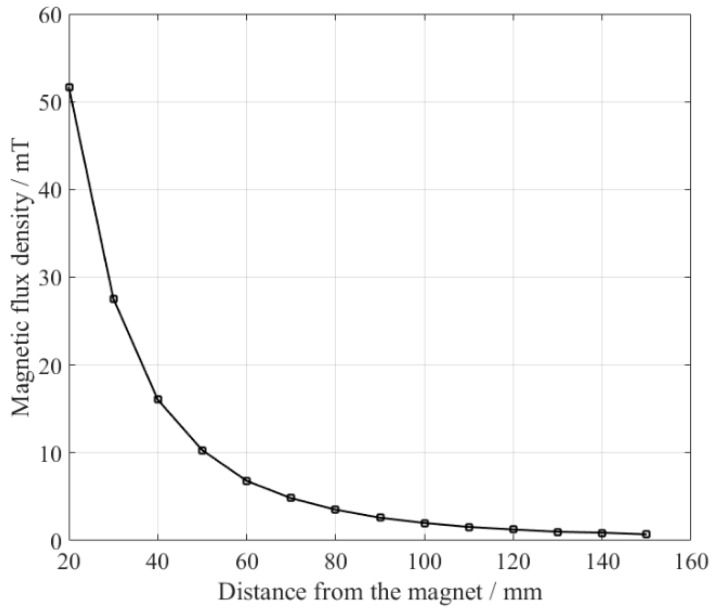
Magnetic flux density at different distances from the permanent magnet.

The optimum lift-offs for the excitation magnet and receiving magnet were 80 mm and 60 mm, respectively, which indicates that the EMAT reaches the highest conversion efficiency when the sample is magnetized at a particular level. Sample’s magnetization could have been calculated from the magnetic field strength and the magnetization curve, but the exact magnetization curve data of the sample was not known. So, the magnetic flux densities at different distances (vertical direction) from the permanent magnet were measured using a gauss meter, and the results are shown in Figure 12. As the distance increases, the magnetic flux density drops sharply. The magnetic flux densities at 80 mm and 60 mm were 3.5 mT and 6.8 mT, respectively. For the excitation EMAT, the dynamic magnetic field was overlapped by the bias magnetic field to excite the ultrasonic wave. The magnetic flux density of the dynamic magnetic field was difficult to be measured due to its higher frequency. So, a three-dimensional FE model was built, and the magnetic field distribution around the excitation coil was simulated (not shown). The results show that the dynamic magnetic flux density at the sample varied from 0 to 27 mT (in the air). 

**Figure 13:**
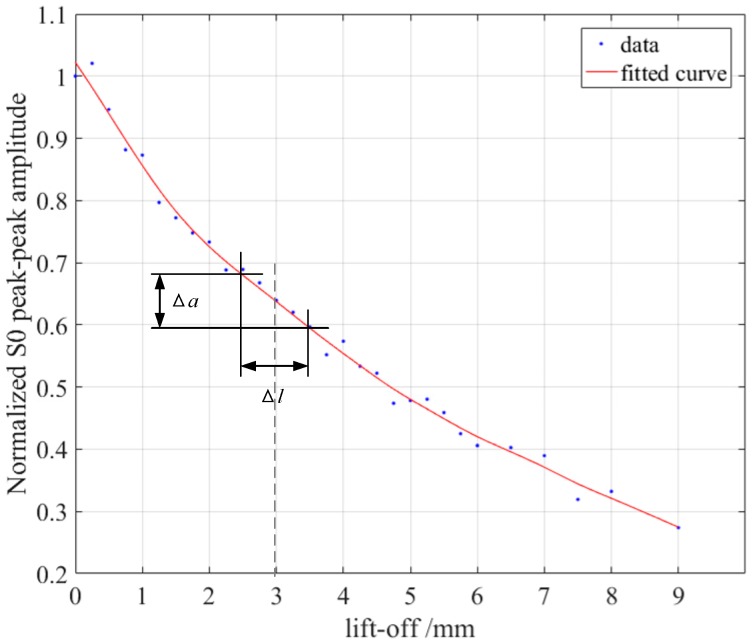
The lift-off of the receiving coil versus the amplitude of the S0 Lamb wave.

The previous study showed that the efficiency of the magnetostriction-based EMAT was robust to the variation of the excitation coil’s lift-off when the lift-off was greater than 5 mm [19]. So, the relationship between the lift-off of the receiving coil and the efficiency was also studied here. The same experiment configuration shown in Figure 7 was applied. In the experiments, we sequentially increased the lift-off of the receiving coil and recorded the waveforms of S0 waves. These S0 waves’ peak-to-peak values were calculated and are shown in Figure 13. They were also fitted in a smooth solid line. The results show that, as the lift-off of the receiving coil increases, the transducer’s transducing efficiency gradually decreases, and the smaller the lift-off is, the faster the efficiency declines.

In online measurement, the distance between the transducer and the sample will inevitably change due to various reasons, such as vibration. Vibration usually causes measurement error, which is evaluated here. It is assumed that the change of the lift-off caused by vibration is fixed, represented as Δl. Then, the variation of the S0 wave’s peak-to-peak value Δa can be acquired from the curve in Figure 13. It is evident that Δa becomes smaller as the lift-off rises, but the S0 wave’s amplitude A also diminishes. So, the relative error is introduced:

(2) $$e=\frac{\Delta a}{A}\;.$$

**Figure 14:**
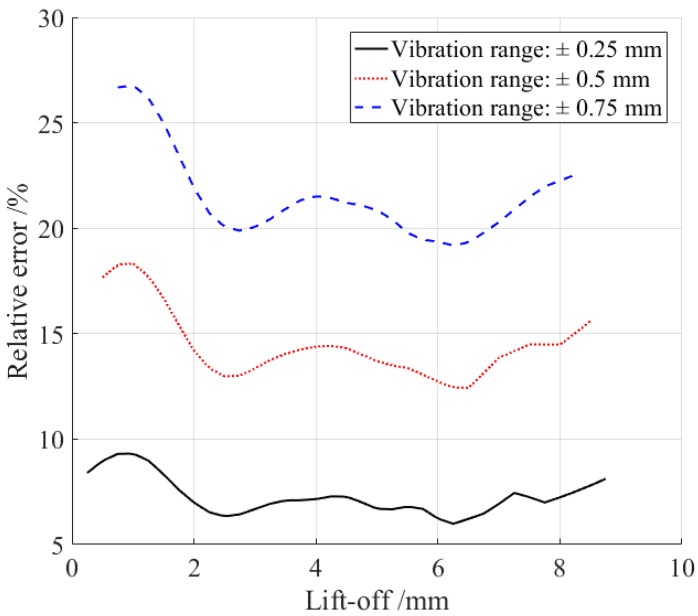
The relative error caused by vibration.

A lower relative error helps to enhance the measurement accuracy. The aim is to ascertain an appropriate lift-off distance to minimize the relative error e. It is assumed that the vibration Δl has several specific values, and the distribution of the vibration is in the upper and lower symmetry range of the lift-off, for example, ±0.25 mm, ±0.5 mm, ±0.75 mm. According to the curve in Figure 13, the relative errors under the three different vibration ranges were calculated, as shown in Figure 14. The results reveal that the relative error rises with the increment of the vibration amplitude. So, the vibration amplitude should be restricted to be as low as possible in the actual measurement. Moreover, the relative error also changes with the lift-off, reaching a maximum at about a 1-mm lift-off, and a minimum at about a 3-mm lift-off. So, the lift-off of the receiving coil was set to 3 mm around to reduce the measurement error. In the practical application, system noise also should be considered to optimize the optimum lift-off. 

## 5. Discussion

The method proposed in this paper does not give a complete magnetostriction curve but can be used for relative measurement, which could be designed to compare the magnetostriction coefficients of two materials. Before the measurement, the reference sample needs to be calibrated. Then, the magnetostriction coefficient of the sample to be tested can be assessed according to the reference. Furthermore, the sample should have the same thickness as the reference. The method also has advantages including non-contact online rapid measurement as well as a continuous measurement process. Moreover, the magnetostriction coefficient of different parts can be given in real-time throughout the moving of the sample. This method provides an alternative to the rapid measurement of the magnetostriction coefficient of silicon steel strips in industrial production.

Three samples with different silicon contents were used in the experiments, which may have induced divergences in both the conductivity and Young’s modulus [32,33]. Thus, their influences on the measurement results are discussed here. For traditional EMATs, the Lorentz force mechanism (generator) and electromagnetic induction mechanism (receiver) are the main contributors to the conversion efficiency, which significantly depend on the sample’s electrical conductivity [18]. However, the magnetostriction effect is not inherently related to the conductivity, although the conductivity may affect the eddy current distribution in the sample. Therefore, the slight decrease in conductivity caused by the increment of the silicon content was neglected. Generally, under the identic dynamic Lorenz force, the amplitude of the ultrasonic is inversely proportional to the Young’s modulus. The magnetostriction was established between the magnetic field and the strain, which is irrelevant to the Young’s modulus. So, the variation of Young’s modulus was also neglected. 

The magnetostriction coefficients of oriented silicon steels are generally anisotropic and related to their orientation, so their orientation needs to be clarified before their measurement. The orientations of all the samples need to be consistent; for example, parallel or perpendicular to the measurement direction. The process of rolling may introduce slight directionality in the magnetostriction of non-oriented silicon steel. So, it is also recommended that the measurement directions of all the samples have the same angle to the rolling direction.

Unfortunately, we were unable to obtain the absolute value of the magnetostriction coefficient of the three samples. Attempts were conducted to measure their magnetostriction coefficients using the strain gauge method, but the measurement results were not reliable due to the low measurement accuracy.

Both stress and temperature have a significant impact on the magnetostriction coefficient [34,35], so the fluctuations of the temperature and stress should be kept in a small range during the measurement. Based on the influence of stress on the magnetostriction coefficient, this method could be further used to measure the stress, and we are researching this at present. The excitation current employed in this paper is a wide-band pulse current, but it can still be replaced by the narrow-band pulse current [36], which is more conducive to denoising by band-pass filtering to obtain a higher SNR. This method is not only capable of measuring a strip-shaped sample, but can also be utilized to measure tubular, block-like specimens by changing the structure of the transducer.

## 6. Conclusions

A 2-D nonlinear magnetostriction finite element model was established to simulate the Lamb S0 waves generated by magnetostriction-based EMAT in silicon steel strips with different magnetostriction coefficients. The simulation results reveal that the magnetostriction coefficient of the sample had a linear relationship with the amplitude of the excited Lamb S0 wave. Measurements were carried out on silicon steel strips with silicon contents of 3%, 4.5%, and 6%. The experimental results show that the magnetostriction-based EMAT can effectively measure their relative magnetostriction coefficients.

The conversion efficiency of the magnetostriction-based EMAT changes with the variation of the magnitude of the bias magnetic field. Experiments were conducted to study their relationship, in which a silicon steel strip with a 3% silicon content was employed as the sample. The results indicate that, as the size of the horizontal bias magnetic field rises, the efficiencies of both the excitation and receiving transducers rise first and then decrease, and reach their maximum efficiency when the magnetic flux densities are 3.5 mT and 6.8 mT, respectively. The experiment results also show that the conversion efficiency drops dramatically with the increase of the lift-off of the receiving coil. The relative error caused by the vibration between the sample and the receiving coil was analyzed, and the relative error was found to reach the minimal size when the lift-off of the receiving coil was set to 3 mm around.
